# Constructing a prognostic model for patients over 70 years of age with unoperated non-small cell lung cancer: A LASSO regression method

**DOI:** 10.1097/MD.0000000000048526

**Published:** 2026-05-08

**Authors:** Feiyang Li, Fang Li, Haowei Lu

**Affiliations:** a Ward 2, Department of Medical Oncology, Lixin People’s Hospital, Bozhou City, Anhui Province, China; b Ward 1, Department of Medical Oncology, Affiliated Hospital of Qinghai University, Xining City, Qinghai Province, China.

**Keywords:** elderly, nomogram, NSCLC, predictive modeling, SEER database

## Abstract

The proportion of elderly patients with non-small cell lung cancer (NSCLC) undergoing surgical treatment is low. However, the prognostic factors influencing the outcomes of nonsurgical patients have not been systematically studied. Therefore, we aim to construct a prognostic prediction model for this patient population to provide a more accurate survival prediction. We conducted a retrospective analysis of patients with pathologically diagnosed NSCLC. We constructed nomograms for overall survival (OS) and cancer-specific survival (CSS) at 1, 3, and 5 years using least absolute shrinkage and selection operator regression and Cox regression analysis. The performance of the predictive models was evaluated using consistency indices, calibration curves, receiver operating characteristic curves, and decision curve analysis. Both internal and external validations were performed. A total of 18,939 NSCLC patients were included, divided into training and validation sets in a 7:3 ratio. The chi-square test indicated no statistically significant difference between the 2 datasets regarding baseline information (*P* > .05). Through least absolute shrinkage and selection operator regression and Cox regression analyses, we identified age, sex, marital status, American Joint Committee on Cancer stage, radiotherapy, chemotherapy, and distant metastasis as influencing factors for OS. We used these factors to construct a nomogram for OS. Similarly, we identified independent prognostic factors affecting CSS, which included sex, American Joint Committee on Cancer stage, radiotherapy, chemotherapy, and distant metastasis, and constructed a nomogram for CSS. After construction, we validated the prognostic models for OS and CSS using receiver operating characteristic curves, consistency indices, calibration curves, and decision curve analysis, which demonstrated the accuracy and reliability of our models. Finally, we confirmed the feasibility of using these models in different populations through external validation sets. This predictive model can provide a more accurate prognostic assessment for non-operated NSCLC patients over 70 years old.

## 1. Introduction

The most recent global cancer statistics published in 2024 indicate that there were 2.5 million new cases of lung malignancies in 2022, which represents 12.4% of all newly diagnosed cancers. Additionally, lung malignancies accounted for 1.8 million deaths in the same year, positioning them as the leading cause of both new cases and mortality among cancers. This underscores the significant threat lung malignancies pose to human health, contributing to a substantial cancer burden.^[1]^ Lung malignancies are primarily classified into 2 pathological types: non-small cell lung cancer (NSCLC) and small-cell lung cancer, with NSCLC being the predominant type, comprising approximately 80% of all lung malignancies.^[2]^ As the global population ages, the incidence of lung malignancies among the elderly is on the rise. Research indicates that individuals aged 65 years and older represent about 70% of all lung malignancy cases, with a median age of diagnosis at 70 years.^[3]^ Currently, NSCLC patients aged 65 and older constitute more than 55% of lung malignancy cases, highlighting the elderly as a significant demographic within this category. Projections based on GLOBOCAN 2020 data suggest that by 2050, the number of new lung cancer cases may reach approximately 3.8 million, with an increasing proportion of elderly patients.^[4,5]^ Elderly patients are particularly vulnerable to high mortality rates due to the presence of comorbidities, diminished organ reserve capacity, and poor nutritional status, necessitating focused attention on this population.^[6]^

There is a paucity of relevant guidelines pertaining to the treatment of elderly patients with tumors, with the National Comprehensive Cancer Network guidelines being the sole organization to formulate established specific recommendations for the treatment management of this patient population. The management of elderly patients is developed in accordance with the guidelines for NSCLC. The therapeutic approaches encompass surgery, chemotherapy, radiotherapy, immunotherapy, and targeted therapies.^[7]^ Numerous prospective clinical trials establish stringent age criteria. One study indicated that the proportion of patients aged 70 years or older in clinical trials focused on the treatment of NSCLC conducted between 1990 and 2012 was approximately 25%.^[8]^ The absence of evidence-based medical research has generated controversy regarding the suitability of certain treatments for older adults, particularly surgical interventions, which tend to exhibit variable efficacy. Furthermore, the proportion of older adults who undergo surgical procedures decreases with advancing age.^[9,10]^ The majority of older adults experience declines in lean body mass, strength, endurance, balance, and walking ability. Research indicates that the extent of these declines is strongly correlated with 30-day, 90-day, and 1-year postsurgical mortality rates in this population. Consequently, surgical interventions necessitate comprehensive preoperative screening for older adults. Such rigorous preoperative assessments are essential, as they can reduce the likelihood of surgical procedures being performed on older patients. As a result, the proportion of older adults undergoing surgical treatment is significantly lower compared with that of younger individuals.^[11]^

In the case of elderly patients with NSCLC who have not undergone surgical intervention, survival prognosis is predominantly assessed using the American Joint Committee on Cancer (AJCC) staging system. While this staging system is widely utilized in clinical practice, it has notable limitations. Specifically, it primarily considers the anatomical features of the tumor and fails to account for the prognostic significance of various demographic factors.^[12]^ Heterogeneity mediated by metabolic and immune resistance mechanisms is also a significant source of variability in the biological behavior of lung cancer. This intratumoral heterogeneity, driven by the interplay between metabolism and immunity, can lead to differing clinical prognoses in patients with the same AJCC stage, resulting in individualized outcome variations between patients.^[13]^ In addition, a search of PubMed revealed a lack of studies addressing the prognostic factors for elderly patients with NSCLC who did not undergo surgical treatment. Consequently, we aimed to investigate the prognostic factors associated with these patients using nomograms. The nomogram is a novel predictive model that integrates a diverse array of common clinical data to develop a prognostic framework that aligns more closely with the various subtypes of lung cancer. This model can be visualized in an intuitive and straightforward manner, thereby facilitating informed clinical decision-making for both patients and healthcare providers. Furthermore, it has gained widespread application in the assessment of various solid tumors.^[14]^ Furthermore, a predictive model was developed in our study utilizing data on elderly patients with NSCLC obtained from the SEER database. This extensive North American tumor registry encompasses approximately 30% of the North American population, thereby offering a diverse and reliable dataset for our research.^[15]^ We aim to develop a precise, efficient, and user-friendly prognostic prediction model for elderly patients diagnosed with NSCLC who have not undergone surgical intervention, utilizing the available data. This model is intended to assist clinicians in more accurately forecasting the prognosis of this patient demographic, thereby providing a foundation for personalized treatment and management strategies. Additionally, it seeks to contribute to the establishment of guidelines pertinent to the care of elderly patients with NSCLC.

## 2. Materials and methods

### 2.1. Data sources and research design

We utilized SEER*Stat version 8.4.3 (Information Management Services, Inc., Calverton) to acquire data on lung malignancies from the release dated April 17, 2024. This dataset encompassed information regarding tumor stage, clinicopathologic characteristics, and survival outcomes. Prior to the commencement of this study, we submitted a data use agreement to the SEER project team and received formal authorization to access the database, as indicated by approval number 14038-Nov2021. The SEER database serves as a resource for public research, ensuring that all personal data contained within is treated with the utmost confidentiality. The dataset utilized in this study has undergone external validation and has been deidentified to safeguard patient privacy. Consequently, ethical approval was not required for this study. The research was conducted in compliance with the Declaration of Helsinki (revised 2013). These data were screened using inclusion and exclusion criteria, which were as follows: age ≥70 years; RX Summ–Surg Prim Site (1998+): 00 (no surgical procedure of primary site); and year of diagnosis from 2010 to 2015. Exclusion criteria were as follows: ICD-O-3 pathologic diagnosis: 8002/3: malignant tumor, small cell type, 8041/3: small cell carcinoma, not otherwise specified, 8043/3: small cell carcinoma, fusiform cell, 8044/3: small cell carcinoma, intermediate cell, 8045/3: combined small cell carcinoma; patients with survival <1 month; and patients with missing critical information.

### 2.2. Research variables

Patients who were screened for the aforementioned conditions were categorized into training and validation sets in a ratio of 7:3, and relevant variables were extracted from the data. The demographic characteristics of the study population encompassed age categories (70–74.9, 75–79.9, and 80+ years), sex (male and female), race (Black, White, and Other races), and marital status (divorced, married, and single). Tumor characteristics included tumor location (left lung and right lung), AJCC stage (I, II, III, and IV), administration of radiation therapy (yes or no), administration of chemotherapy (yes or no), and details regarding distant metastases (bone metastases, brain metastases, and liver metastases). The data also included information regarding the patients’ survival duration, survival status, and the cause of death.

### 2.3. Statistical analysis

The demographic characteristics and tumor-related information of the training and validation sets were compared using the chi-square test. The prognostic influences of overall survival (OS) and cancer-specific survival (CSS) were assessed through least absolute shrinkage and selection operator (LASSO) regression, and the identified factors were integrated into the Cox model to further determine independent prognostic influences affecting OS and CSS. Prognostic prediction models were constructed using these factors and represented in nomograms, which facilitated the prediction of OS and CSS survival rates at 1, 3, and 5 years. We employed the consistency indices (C-index) to evaluate the discriminatory power of the model. The thresholds for the C-index, which indicate the prediction accuracy represented in the nomogram, were categorized as follows: low accuracy (0.50–0.70), medium accuracy (0.71–0.90), and high accuracy (>0.90).^[16]^ The model was assessed utilizing the receiver operating characteristic (ROC) curve and the area under the curve (AUC), and an AUC value ranging from 0.50 to 0.70 was classified as exhibiting low accuracy, a range of 0.71 to 0.90 was deemed moderately accurate, and values exceeding 0.90 were regarded as highly accurate.^[17]^ The calibration of the model was evaluated by plotting calibration curves utilizing the bootstrap method, which involved 1000 repetitions of sampling to ensure the model’s accuracy. The decision curve analysis (DCA) was conducted to evaluate the clinical benefit and applicability of the model. Subsequently, we validated the constructed model both internally, utilizing the validation set, and externally, employing an external cohort. All statistical analyses were executed using R software (version 4.4.1; R Foundation for Statistical Computing, Vienna, Austria), with a *P* value of <.05 deemed indicative of a statistically significant difference in this study.

## 3. Results

### 3.1. Patient characteristics

**Figure 1.**
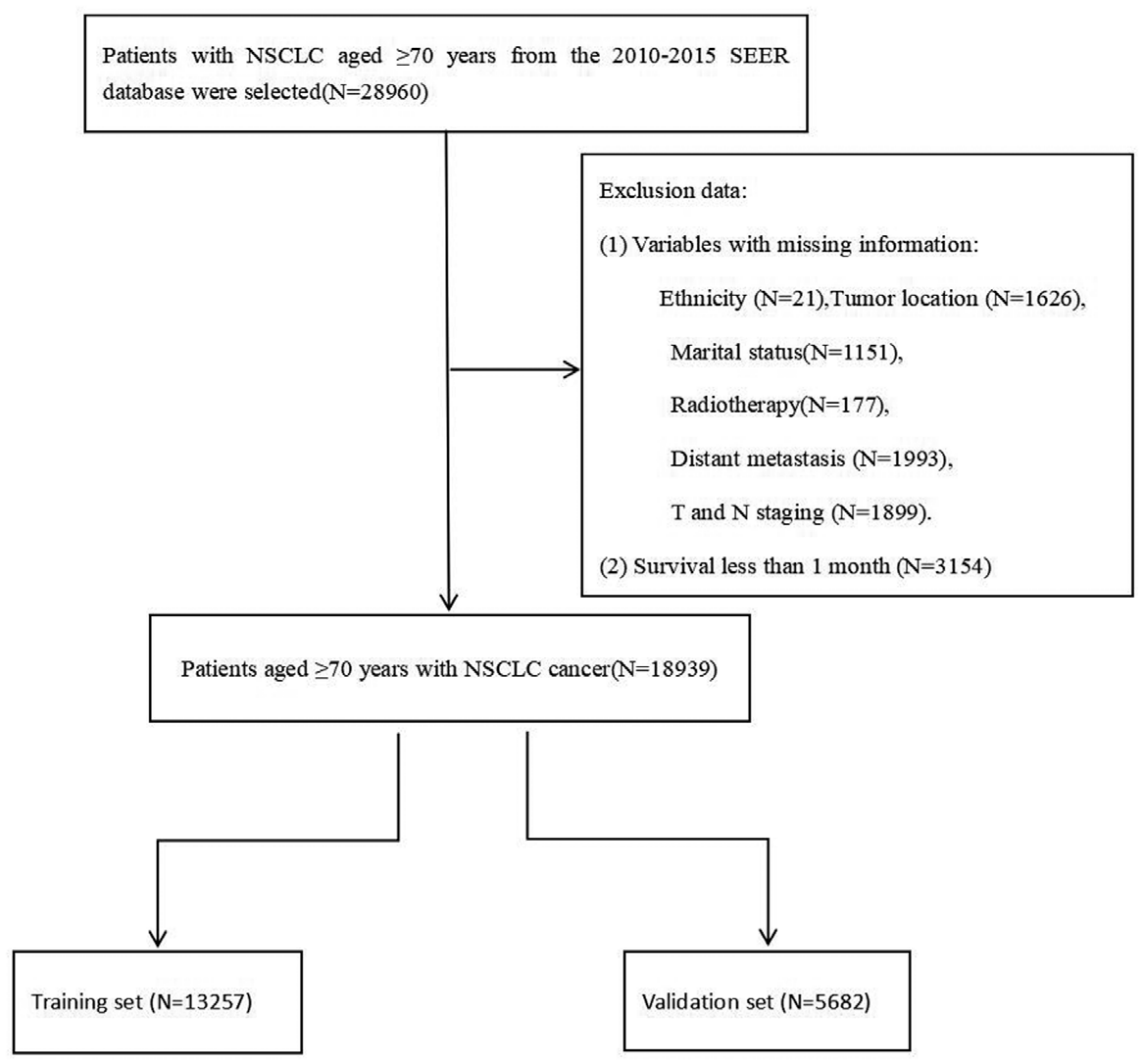
Workflow diagram. NSCLC = non-small cell lung cancer.

In this study, a total of 28,960 patients diagnosed with NSCLC and aged 70 years or older, who had not undergone surgical intervention, were screened from the SEER database. Among these, 18,939 patients met the inclusion criteria and did not meet the exclusion criteria. These patients were then randomly divided into a training set (N = 13,257) and a validation set (N = 5682) using a 70:30 randomization ratio, as illustrated in Figure 1. The largest proportion of patients in the training and validation cohorts was aged 80 years or older, accounting for approximately 42% of the sample. The sex distribution was nearly equal, with both men and women comprising about 50% of the population. The racial composition of the training and validation sets was predominantly Caucasian, with this group representing over 80% of the participants. Regarding marital status, approximately 49% of patients were married at the time of diagnosis, while around 30% were divorced. The proportion of patients classified under AJCC stages III and IV was approximately 68%, indicating that a significant majority of elderly patients with NSCLC presented with advanced stages at the time of diagnosis. In terms of treatment modalities, about 53% of elderly NSCLC patients received radiation therapy, whereas the proportion undergoing systemic chemotherapy was approximately 38%. This lower rate of systemic chemotherapy may be attributed to the adverse reactions associated with the treatment. Concerning distant metastasis, the incidence of liver and brain metastases was both <10%, while bone metastasis was observed in approximately 17% of patients, making it the most prevalent type of distant metastasis in NSCLC. The baseline characteristics of the 2 patient groups were comparable, with no statistically significant differences observed (*P* > .05; Table 1).

**Table 1:** Baseline patient information.

Variable	Total (n = 18,939)	Training (n = 13,257)	Validation (n = 5682)	χ^2^	*P*
Age, n (%)				0.25	.882
70–74.9 yr	5447 (28.76)	3804 (28.69)	1643 (28.92)		
75–79.9 yr	5399 (28.51)	3793 (28.61)	1606 (28.26)		
80+ yr	8093 (42.73)	5660 (42.69)	2433 (42.82)		
Sex, n (%)				0.00	.968
Female	9207 (48.61)	6446 (48.62)	2761 (48.59)		
Male	9732 (51.39)	6811 (51.38)	2921 (51.41)		
Race, n (%)				3.68	.159
Black	1249 (6.59)	861 (6.49)	388 (6.83)		
Other*	2105 (11.11)	1509 (11.38)	596 (10.49)		
White	15,585 (82.29)	10,887 (82.12)	4698 (82.68)		
Marital status, n (%)				1.18	.555
Married	9404 (49.65)	6614 (49.89)	2790 (49.10)		
Single	3701 (19.54)	2569 (19.38)	1132 (19.92)		
Widowed	5834 (30.80)	4074 (30.73)	1760 (30.98)		
Laterality, n (%)				0.00	.980
Left	7937 (41.91)	5555 (41.90)	2382 (41.92)		
Right	11,002 (58.09)	7702 (58.10)	3300 (58.08)		
AJCC stage group, n (%)				1.95	.582
I	4401 (23.24)	3071 (23.17)	1330 (23.41)		
II	1570 (8.29)	1123 (8.47)	447 (7.87)		
III	4231 (22.34)	2961 (22.34)	1270 (22.35)		
IV	8737 (46.13)	6102 (46.03)	2635 (46.37)		
Radiation, n (%)				0.26	.610
No	8786 (46.39)	6134 (46.27)	2652 (46.67)		
Yes	10,153 (53.61)	7123 (53.73)	3030 (53.33)		
Chemotherapy, n (%)				1.36	.244
No	11,590 (61.20)	8077 (60.93)	3513 (61.83)		
Yes	7349 (38.80)	5180 (39.07)	2169 (38.17)		
Bone metastasis, n (%)				0.03	.871
No	15,702 (82.91)	10,995 (82.94)	4707 (82.84)		
Yes	3237 (17.09)	2262 (17.06)	975 (17.16)		
Brain metastasis, n (%)				3.79	.051
No	17,139 (90.50)	11,961 (90.22)	5178 (91.13)		
Yes	1800 (9.50)	1296 (9.78)	504 (8.87)		
Liver metastasis, n (%)				0.19	.665
No	17,566 (92.75)	12,303 (92.80)	5263 (92.63)		
Yes	1373 (7.25)	954 (7.20)	419 (7.37)		

AJCC = American Joint Committee on Cancer.

* American Indian/AK Native, Asian/Pacific Islander.

### 3.2. Independent prognostic factors for OS and CSS

**Figure 2.**
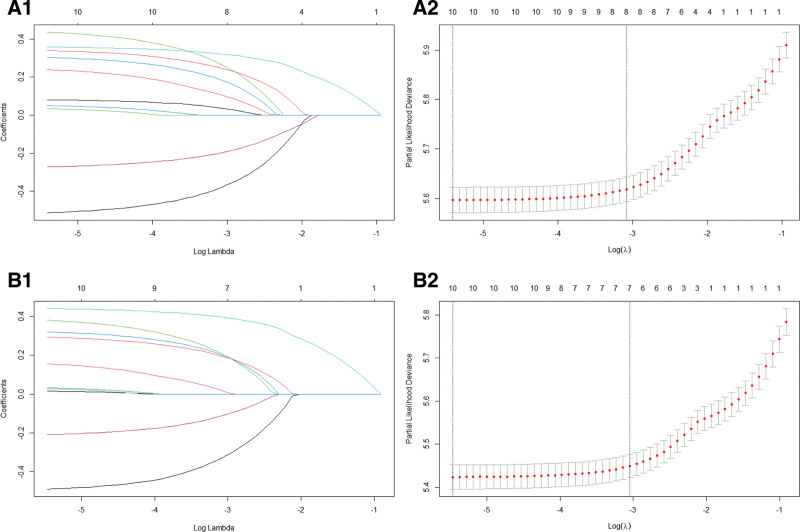
A1 is the LASSO coefficient of 10 features that affect OS, A2 is the selection of the tuning parameter (k) for the LASSO model; B1 is the LASSO coefficient of 10 features that affect CSS, B2 is the selection of the tuning parameter (k) for the LASSO model. CSS = cancer-specific survival, LASSO = least absolute shrinkage and selection operator, OS = overall survival.

We employed LASSO regression on the variables within the training dataset, utilizing the glmnet function with the family parameter configured to Cox. Following the application of the compression algorithm, the regression coefficients for the majority of the variables were reduced to 0 (Fig. 2A1). The mean square error was plotted utilizing 10-fold cross-validation as the natural logarithm of the lambda (λ) parameter varied. The mean square error reached its minimum value when λ was reduced to 0.005, as indicated by the left dashed line. The right dashed line represents the value of λ that falls within 1 standard error (Fig. 2A2). The 10 variables identified with nonzero coefficients include age, sex, race, marital status, AJCC stage, radiotherapy, chemotherapy, bone metastasis, brain metastasis, and liver metastasis. The incorporation of these variables into the Cox univariate analysis indicated that age, sex, marital status, AJCC staging, radiotherapy, chemotherapy, bone metastasis, brain metastasis, and liver metastasis may serve as prognostic factors for OS (*P* < .1). Subsequently, these factors were included in the Cox multivariate analysis, which demonstrated that age, sex, marital status, AJCC staging, radiotherapy, chemotherapy, bone metastasis, brain metastasis, and liver metastasis were independent prognostic factors for OS (*P* < .05; Table 2).

**Table 2:** One-factor and multi-factor Cox analysis of OS in the training set.

Variate	Univariate analysis			Multivariate analysis		
	*P*	HR	95% CI	*P*	HR	95% CI
Age						
70–74.9 yr	Reference			Reference		
75–79.9 yr	.005	1.07	1.02–1.12	.002	1.08	1.03–1.13
80+ yr	<.001	1.16	1.12–1.22	<.001	1.20	1.14–1.25
Sex						
Female	Reference			Reference		
Male	<.001	1.23	1.19–1.28	<.001	1.31	1.31–1.36
Race						
Black	Reference			–	–	–
Other*	.257	0.95	0.87–1.04	–	–	–
White	.188	0.95	0.89–1.02	–	–	–
Marital status						
Married	Reference			Reference		
Single	.270	1.03	0.98–1.08	<.001	1.12	1.06–1.17
Widowed	.058	1.04	1.00–1.08	<.001	1.14	1.09–1.19
AJCC stage group						
I	Reference			Reference		
II	<.001	1.70	1.58–1.82	<.001	1.91	1.78–2.06
III	<.001	1.73	1.64–1.83	<.001	2.33	2.20–2.47
IV	<.001	3.03	2.88–3.17	<.001	3.04	2.86–3.23
Radiation						
No	Reference			Reference		
Yes	<.001	0.62	0.60–0.65	<.001	0.76	0.73–0.79
Chemotherapy						
No	Reference			Reference		
Yes	.020	0.96	0.92–0.99	<.001	0.59	0.56–0.61
Bone metastasis						
No	Reference			Reference		
Yes	<.001	2.16	2.06–2.26	<.001	1.50	1.42–1.59
Brain metastasis						
No	Reference			Reference		
Yes	<.001	2.08	1.96–2.21	<.001	1.55	1.45–1.65
Liver metastasis						
No	Reference			Reference		
Yes	<.001	2.26	2.11–2.42	<.001	1.37	1.28–1.47

AJCC = American Joint Committee on Cancer, CI = confidence interval, HR = hazard ratio, OS = overall survival.

* American Indian/AK Native, Asian/Pacific Islander.

We screened for prognostic factors that may influence CSS utilizing LASSO regression. The factors considered included age, sex, race, marital status, AJCC stage, radiotherapy, chemotherapy, and the presence of bone, brain, and liver metastases (Fig. 2B1 and B2). The integration of these factors into both univariate and multivariate Cox analyses indicated that sex, AJCC stage, radiotherapy, chemotherapy, and the presence of bone, brain, and liver metastases were identified as independent prognostic factors for CSS (*P* < .05; Table 3).

**Table 3:** One-factor and multi-factor Cox analysis of CSS in the training set.

Variate	Univariate analysis			Multivariate analysis		
	*P*	HR	95% CI	*P*	HR	95% CI
Age						
70–74.9 yr	Reference			–	–	–
75–79.9 yr	.634	0.99	0.94–1.04	–	–	–
80+ yr	.386	0.98	0.93–1.03	–	–	–
Sex						
Female	Reference			Reference		
Male	<.001	1.13	1.08–1.17	<.001	1.13	1.09–1.18
Race						
Black	Reference			–	–	–
Other*	.426	1.04	0.94–1.15	–	–	–
White	.694	0.98	0.91–1.07	–	–	–
Marital status						
Married	Reference			–	–	–
Single	.285	0.97	0.92–1.02	–	–	–
Widowed	.449	0.98	0.94–1.03	–	–	–
AJCC stage group						
I	Reference			Reference		
II	<.001	1.94	1.77–2.12	<.001	1.93	1.76–2.11
III	<.001	2.20	2.06–2.36	<.001	2.17	2.03–2.32
IV	<.001	3.95	3.72–4.20	<.001	2.89	2.70–3.09
Radiation						
No	Reference			Reference		
Yes	<.001	0.63	0.60–0.65	<.001	0.73	0.70–0.76
Chemotherapy						
No	Reference			–	–	–
Yes	.454	1.02	0.98–1.06	–	–	–
Bone metastasis						
No	Reference			Reference		
Yes	<.001	2.18	2.07–2.29	<.001	1.33	1.25–1.40
Brain metastasis						
No	Reference			Reference		
Yes	<.001	2.16	2.03–2.30	<.001	1.54	1.44–1.65
Liver metastasis						
No	Reference			Reference		
Yes	<.001	2.43	2.27–2.61	<.001	1.40	1.31–1.51

AJCC = American Joint Committee on Cancer, CI = confidence interval, CSS = cancer-specific survival, HR = hazard ratio.

* American Indian/AK Native, Asian/Pacific Islander.

### 3.3. Construction of OS and CSS prognostic nomograms

**Figure 3.**
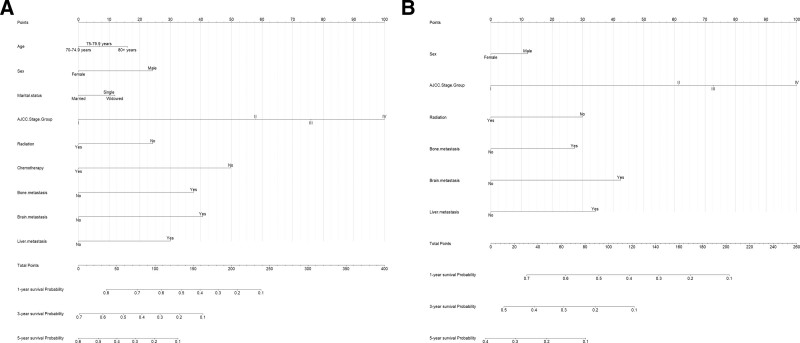
Prognostic nomograms of 1-, 3-, and 5-year OS (A) and CSS (B). CSS = cancer-specific survival, OS = overall survival.

We developed nomograms for OS and CSS by incorporating the independent factors identified through LASSO regression into the Cox regression analysis. These nomograms were utilized to predict patient survival by integrating all relevant predictors. The total score, derived from the summation of the scores assigned to each variable, was used to forecast the 1-, 3-, and 5-year survival rates of patients (Fig. 3).

### 3.4. Validation of nomograms

**Figure 4.**
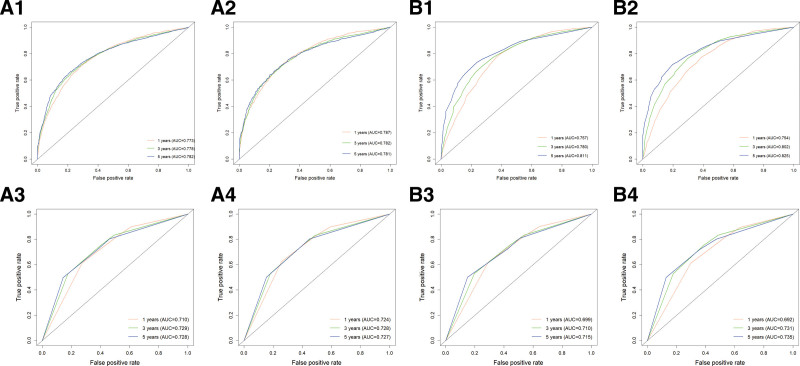
A1 is the OS training set ROC curve, A2 is the OS validation set ROC curve, A3 is the AJCC staging training set ROC curve, A4 is the AJCC staging validation set ROC curve (OS); B1 is the CSS training set ROC curve, B2 is the CSS validation set ROC curve, B3 is the AJCC staging training set ROC curve, B4 is the AJCC staging validation set ROC curve (CSS). AJCC = American Joint Committee on Cancer, AUC = area under the curve, CSS = cancer-specific survival, OS = overall survival, ROC = receiver operating characteristic.

**Figure 5.**
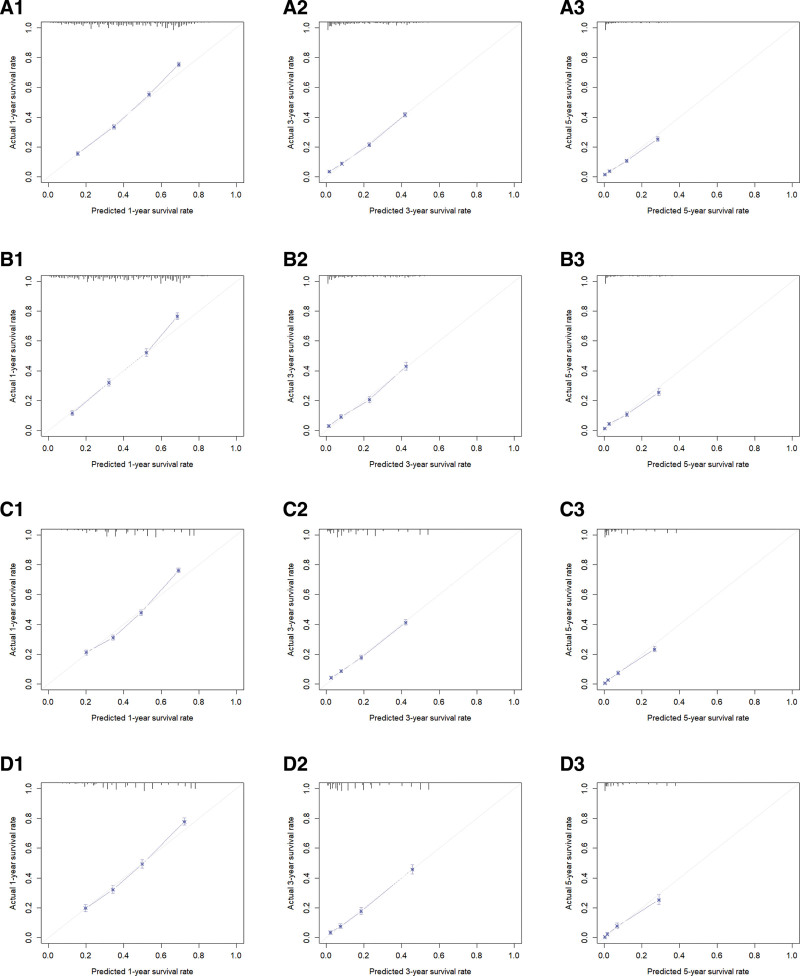
A1, A2, and A3 are 1-, 3-, and 5-year calibration curves of the OS training set, respectively; B1, B2, and B3 are 1-, 3-, and 5-year calibration curves of the OS validation set, respectively; C1, C2, and C3 are the 1-, 3-, and 5-year calibration curves of the CSS training set, respectively; D1, D2, and D3 are the 1-, 3-, and 5-year calibration curves of the CSS validation set, respectively. CSS = cancer-specific survival, OS = overall survival.

Utilizing R software, the C-indices were determined to be 0.707 and 0.715 for the OS training and validation sets, respectively, and 0.696 and 0.694 for the CSS training and validation sets, respectively. These results suggest that the model demonstrates a commendable predictive value (Table 4). In the R software environment, risk scores for each independent prognostic factor were computed, and ROC curves for 1-, 3-, and 5-year OS and CSS were generated for the training sets. These findings were subsequently validated using the validation set by plotting ROC curves for the same time intervals. In the OS training set, the AUC values for 1-, 3-, and 5-year intervals were 0.773, 0.778, and 0.782, respectively. In contrast, the AUC values for the OS validation set for the same intervals were 0.787, 0.782, and 0.781. The AUC values for the CSS training set were 0.757, 0.780, and 0.811 for 1, 3, and 5 years, respectively. In comparison, the AUC values for the CSS validation set were 0.754, 0.802, and 0.825 for the same time intervals (Fig. 4). The results presented above demonstrate the capability of this predictive model to reliably assess the OS and CSS rates at 1, 3, and 5 years for unoperated elderly patients with NSCLC. Subsequently, the model underwent validation utilizing the bootstrap method, with a sampling number set at *B* = 1000. The validation outcomes indicated that the survival correction curves for OS and CSS at 1, 3, and 5 years closely aligned with the 45° reference line, suggesting a high degree of accuracy in the predictive performance (Fig. 5).

**Table 4:** C-index results.

	C-index	SE (C-index)
OS		
Training set	0.707	0.002
Validation set	0.715	0.003
AJCC	0.650	0.002
CSS		
Training set	0.696	0.002
Validation set	0.694	0.004
AJCC	0.639	0.002

AJCC = American Joint Committee on Cancer, C-index = consistency indices, CSS = cancer-specific survival, OS = overall survival, SE = standard error.

### 3.5. DCA analysis

**Figure 6.**
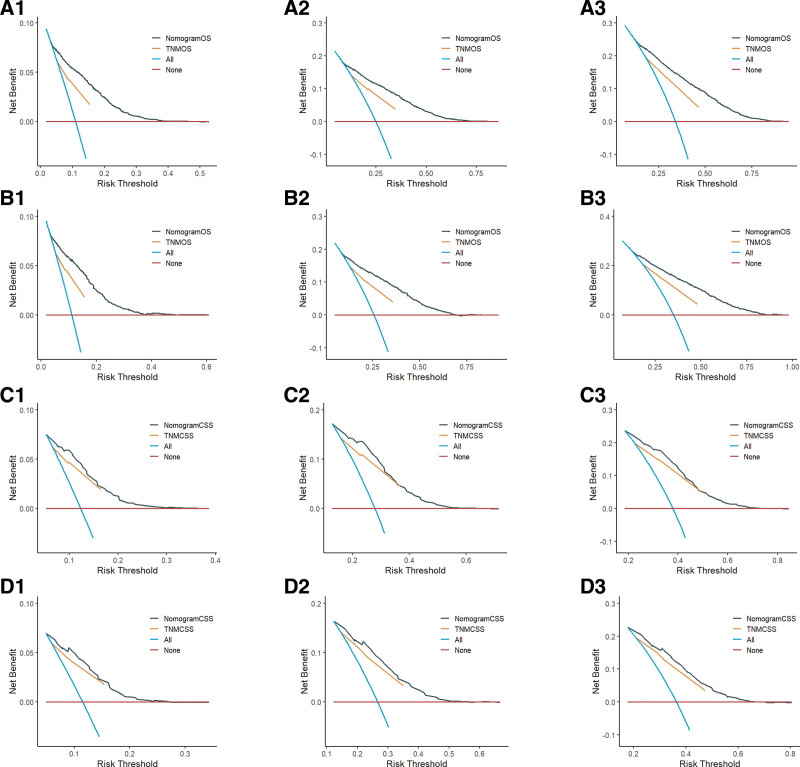
A1, A2, and A3 are the 1-, 3-, and 5-year DCA curves for the OS training set; B1, B2, and B3 are the 1-, 3-, and 5-year DCA curves for the OS validation set; C1, C2, and C3 are the 1-, 3-, and 5-year DCA curves for the CSS training set; D1, D2, and D3 are the 1-, 3-, and 5-year DCA curves for the CSS validation set, respectively. CSS = cancer-specific survival, DCA = decision curve analysis, OS = overall survival, TNMCSS = tumor, node, metastasis cancer-specific survival, TNMOS = tumor, node, metastasis overall survival.

DCA is an assessment method utilized to evaluate the extent of patient benefit. The area under the ROC curve solely measures the diagnostic accuracy of a predictive model, neglecting the clinical utility of that model. This oversight may result in overmedication, a concern that can be effectively addressed through DCA curves.^[18]^ In the present study, DCA curves were generated based on the 1-, 3-, and 5-year survival rates of patients in the OS and CSS prognostic models. Research findings suggest that, in comparison to the AJCC staging system, the 2 models developed in this study offer enhanced clinical net benefits at 1, 3, and 5 years (see Fig. 6).

### 3.6. Comparison with the AJCC staging system

We utilized the AJCC staging system derived from the SEER database to develop models for OS and CSS. The C-indices for the 2 models were determined to be 0.650 and 0.639, respectively (Table 4). Additionally, we generated ROC curves for OS and CSS, yielding AUC values of 0.710, 0.729, and 0.728 for 1, 3, and 5 years in the OS training set. The OS validation set exhibited AUC values of 0.724, 0.728, and 0.727 for the same time intervals. For the CSS training set, the AUC values were 0.699, 0.710, and 0.715 for 1, 3, and 5 years, respectively, while the CSS validation set demonstrated AUC values of 0.692, 0.731, and 0.735 for the corresponding years. Notably, the accuracy of the AJCC staging model was inferior to that of the model we developed (Fig. 4). Furthermore, we illustrated the DCA curves for OS and CSS, revealing that the net clinical benefit rate associated with AJCC staging was significantly lower than that of our constructed model (Fig. 6). Consequently, our model provides a more precise prediction of prognosis for unoperated elderly patients with NSCLC compared with the AJCC staging system.

### 3.7. External validation

**Figure 7.**
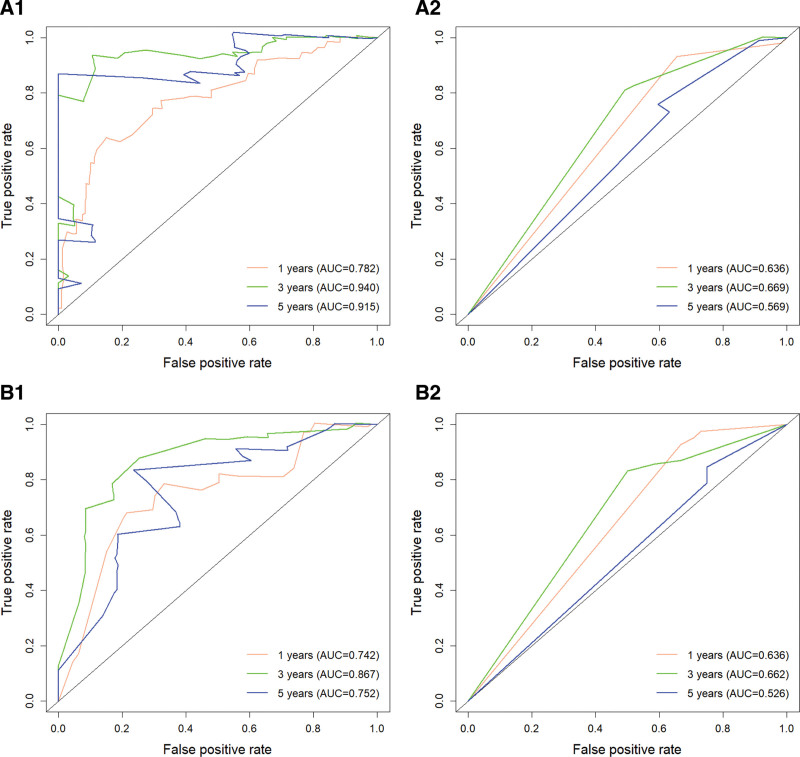
A1 is the ROC curve for the external validation set OS for 1, 3, and 5 years, A2 is the ROC curve for the external validation set AJCC staging for 1, 3, and 5 years (OS); B1 is the ROC curve for the external validation set CSS for 1, 3, and 5 years, B2 is the ROC curve for the external validation set AJCC staging for 1, 3, and 5 years (CSS). AJCC = American Joint Committee on Cancer, AUC = area under the curve, CSS = cancer-specific survival, OS = overall survival, ROC = receiver operating characteristic.

**Figure 8.**
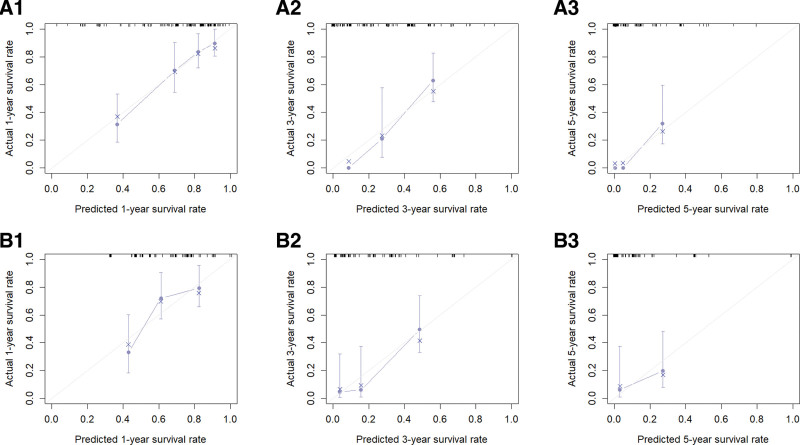
A1, A2, and A3 are the 1-, 3-, and 5-year calibration curves for the external validation set OS; B1, B2, and B3 are the 1-, 3-, and 5-year calibration curves for the external validation set CSS, respectively. CSS = cancer-specific survival, OS = overall survival.

**Figure 9.**
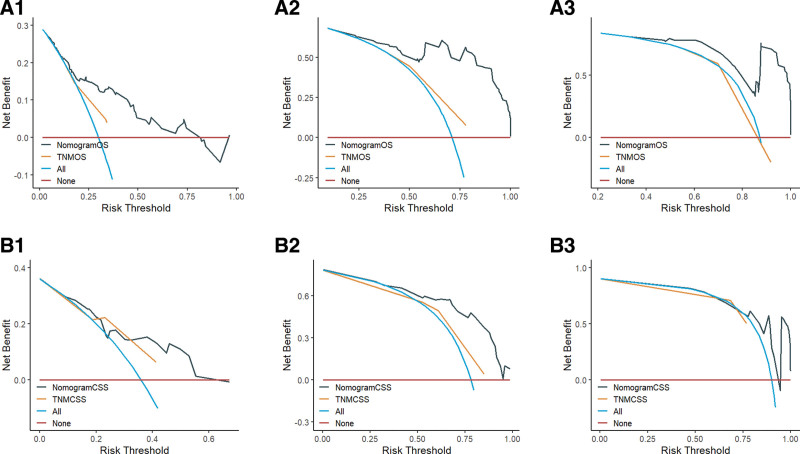
A1, A2, and A3 are the 1-, 3-, and 5-year DCA curves for the external validation set OS; B1, B2, and B3 are the 1-, 3-, and 5-year DCA curves for the external validation set CSS, respectively. CSS = cancer-specific survival, DCA = decision curve analysis, OS = overall survival.

We collected data from NSCLC patients who were pathologically diagnosed at Lixin County People’s Hospital between January 1, 2018, and December 31, 2024, to establish the external validation cohort. To ensure the quality of the study, we selected data based on predefined inclusion and exclusion criteria. The inclusion criteria were as follows: age ≥70 years and no prior surgical treatment. The exclusion criteria were as follows: survival duration <1 month and missing key information. In total, we collected data from 149 NSCLC patients who met the criteria for the external validation cohort. The baseline characteristics of the external validation cohort are presented in Table 5. The final validation results indicated a C-index of 0.724 for OS and 0.685 for CSS, as presented in Table 6. The AUC values for OS at 1, 3, and 5 years were 0.782, 0.940, and 0.915, respectively, while the AUC values for CSS at the same time points were 0.742, 0.867, and 0.752, respectively, as illustrated in Figure 7. The calibration curves of the model were validated using external data, demonstrating that the 1-, 3-, and 5-year survival calibration curves for both OS and CSS closely aligned with the 45° reference line, as shown in Figure 8. Additionally, DCA curves were generated using external data, revealing a higher net clinical benefit at 1, 3, and 5 years, as depicted in Figure 9. Furthermore, we developed a model based on AJCC staging using data from the external validation set for comparative analysis with our model. The model constructed from the external validation set also demonstrated superior performance compared with the AJCC staging. Therefore, through the validation with external data, we have established the applicability of our model across diverse populations.

**Table 5:** Baseline data of the external validation set.

	C-index	SE (C-index)
OS		
Nomogram	0.724	0.032
AJCC	0.592	0.024
CSS		
Nomogram	0.685	0.040
AJCC	0.613	0.025

AJCC = American Joint Committee on Cancer, C-index = consistency indices, CSS = cancer-specific survival, OS = overall survival, SE = standard error.

**Table 6:** External validation set C-index.

Variables	Total (n = 149)
Age, n (%)	
70–74.9 yr	76 (51.01)
75–79.9 yr	48 (32.21)
80+ yr	25 (16.78)
Sex, n (%)	
Female	34 (22.82)
Male	115 (77.18)
Marital status, n (%)	
Married	135 (90.60)
Single	9 (6.04)
Widowed	5 (3.36)
Race, n (%)	
Other	149 (100.00)
Laterality, n (%)	
Left	68 (45.64)
Right	81 (54.36)
AJCC stage group, n (%)	
I	3 (2.01)
II	3 (2.01)
III	36 (24.16)
IV	107 (71.81)
Chemotherapy, n (%)	
No	48 (32.21)
Yes	101 (67.79)
Radiation, n (%)	
No	105 (70.47)
Yes	44 (29.53)
Bone metastasis, n (%)	
No	108 (72.48)
Yes	41 (27.52)
Brain metastasis, n (%)	
No	122 (81.88)
Yes	27 (18.12)
Liver metastasis, n (%)	
No	139 (93.29)
Yes	10 (6.71)

AJCC = American Joint Committee on Cancer, C-index = consistency indices.

## 4. Discussion

Data on lung cancer released by the SEER database on April 17, 2024, indicate that approximately 50% of patients with NSCLC are aged 70 years or older. This percentage is expected to gradually increase in conjunction with the progression of an aging society.^[4,5]^ Elderly patients with lung cancer exhibit a lower rate of surgical intervention, which can be attributed to a higher prevalence of comorbidities and diminished physiological function. Additionally, tumors in older patients often demonstrate a more insidious biological behavior, and early symptoms may be less pronounced. Consequently, this leads to diagnosis at more advanced stages of the disease, resulting in the forfeiture of surgical options for many elderly lung cancer patients who otherwise maintain good general health status.^[19–21]^ Due to the aforementioned factors, there has been an increase in the proportion of elderly patients with NSCLC who do not undergo surgical intervention. The prognosis for these patients differs from that of those who receive surgical treatment; however, there is a lack of studies examining the factors that influence the prognosis of this specific patient population. In light of the aforementioned reasons, we utilized data from the SEER database to develop nomograms for OS and CSS. We demonstrated the predictive value and net clinical benefit of these nomograms through the use of C-indices, ROC curves, calibration curves, and DCA curves. Furthermore, we performed an external validation to assess the feasibility and accuracy of applying these nomograms across diverse populations. LASSO regression is a machine learning technique that systematically shrinks regression coefficients by introducing a penalty function within the traditional Cox regression framework. This method allows for continuous variable selection and optimizes the model to address multicollinearity and overfitting issues.^[22,23]^ In this study, we selected LASSO regression over traditional Cox regression to construct a prognostic model for patients aged 70 years and above with NSCLC who had not undergone surgery, based on the following considerations. First, our dataset includes multiple clinical variables (e.g., age, marital status, and treatment modalities) that may exhibit collinearity. LASSO regression mitigates this issue by shrinking the coefficients of less important variables toward 0, effectively resolving multicollinearity and preventing coefficient overestimation, a common issue in traditional regression methods. Second, LASSO regression performs automatic variable selection by shrinking the coefficients of irrelevant variables to 0, which not only enhances the model’s interpretability but also results in a more concise model compared with traditional stepwise selection methods, where too many variables may be retained, thereby increasing the risk of overfitting. Third, by integrating LASSO with the Cox proportional hazards model (i.e., LASSO-Cox model), we retain the semiparametric advantages of the Cox model while analyzing time-dependent survival data, without assuming constant hazard ratios. However, we are also fully aware of the inherent limitations of LASSO regression: its performance may deteriorate when sample sizes are small or extreme collinearity exists, and when 2 variables are highly correlated, LASSO tends to arbitrarily exclude 1 variable, rather than retaining both, which could lead to the omission of variables with shared prognostic information. Nevertheless, given the large sample size in this study (n = 18,939), which effectively mitigates concerns regarding small-sample performance, and the fact that constructing a nomogram with fewer variables and enhanced clinical interpretability aligns more closely with practical clinical needs compared with ridge regression (which does not perform variable selection but retains all variables while shrinking their coefficients), we concluded that LASSO regression was the most suitable method for this study. Our validation results (C-index for OS in the training set: 0.707, C-index for CSS: 0.696) confirm that this method successfully constructed a robust and clinically applicable prognostic model for this patient population, which previously lacked systematic research.

In our study, age was identified as a prognostic factor influencing OS in elderly patients with unoperated NSCLC. A previous epidemiological survey conducted by Li et al^[24]^ demonstrated a positive correlation between mortality and age in patients with lung cancer, which aligns with the findings of our study. In our study, we observed an 8% increase in mortality among patients aged 75 to 79.9 years and a 20% increase in mortality among patients aged 80 years and older, in comparison to patients aged 70 to 74.9 years. However, this correlation was associated with OS rather than CSS, which is inconsistent with findings from previous studies. It is hypothesized that the observed phenomenon may be attributed to the fact that, with advancing age, patients tend to present with a greater number of comorbid underlying conditions and poorer performance status (PS) scores. These factors are associated with an increased risk of mortality, which subsequently impacts the OS duration of patients.^[6]^ Aging is primarily characterized by the expression of the tumor suppressor genes p16 and p53, along with telomere shortening. The upregulation of p16 and p53 expression in elderly individuals is associated with a slower progression of malignant tumors and a reduced incidence of malignancies in this population.^[25]^ It is plausible that the 2 aforementioned factors contributed to a reduced OS time for the patients, while CSS time remains unaffected by age, as it exclusively accounts for deaths attributable to cancer. Additionally, it is noteworthy that marital status serves as a determinant of OS time but does not influence CSS time, which contrasts with findings from some prior studies. A prospective cohort study involving 500,000 individuals from China indicated that single or divorced individuals exhibited a higher likelihood of developing cardiovascular disease and chronic obstructive pulmonary disease. Furthermore, the study suggested that the increased prevalence of underlying health conditions may shorten OS time in patients; however, it does not appear to influence CSS time.^[26]^ Therefore, this could be the reason why marital status affects OS time but not CSS time. Furthermore, sex was found to influence both OS and CSS, with male patients exhibiting an increased risk of mortality by 23% and 13%, respectively, in comparison to female patients. These findings are consistent with those of a prior cohort study conducted in Sweden by Wheatley-Price et al,^[27]^ which also reported a higher mortality risk among male patients, particularly within the adenocarcinoma subgroup. Our study corroborates these results. Female lung cancer patients exhibit elevated levels of immune cell infiltration. Specifically, the presence of immune cells, including CD3+ T cells and CD8+ T cells, is correlated with improved clinical outcomes across various malignancies. This phenomenon may contribute to the more favorable prognosis observed in female patients.^[28]^

The influence of tumor characterization information on prognosis is well-established. The AJCC staging system, which is based on anatomical factors, plays a crucial role in therapeutic decision-making and prognostic assessment of lung cancer. It is currently the most widely utilized staging system in clinical practice.^[29]^ Our study demonstrates the prognostic significance of AJCC staging in unoperated elderly patients with NSCLC, particularly among those in stages III and IV, where the mortality rate is observed to be double that of patients in stage I. Despite the substantial role of AJCC staging, it is important to acknowledge its limitations; the system primarily emphasizes anatomical factors while neglecting the prognostic implications of various demographic characteristics. This oversight is one of the key motivations behind the development of our model.^[12]^ Radiotherapy is predominantly utilized for this cohort of non-operated patients, with at least 50% of lung cancer patients requiring radiotherapy – whether radical or palliative – throughout the entirety of their treatment. The prognostic benefits of radiotherapy for lung cancer patients are well-established.^[30]^ In our study, radiotherapy was found to reduce CSS-related mortality by 27%, surpassing the OS-related reduction of 24%. This discrepancy may be attributed to the adverse effects of radiotherapy, which likely led to a shorter time to OS for the patients. Nevertheless, this phenomenon did not negate the benefits that patients derived from radiotherapy. Chemotherapy represents a critical systemic treatment modality for malignant tumors and has demonstrated considerable efficacy in elderly patients diagnosed with NSCLC.^[31]^ The results from the IFCT-0501 study indicated that elderly patients with NSCLC could benefit from a 2-agent chemotherapy regimen that includes cisplatin, which was associated with a 36% reduction in the risk of mortality compared with single-agent chemotherapy (hazard ratio = 0.64, 95% confidence interval: 0.52–0.78).^[32]^ While our findings are consistent with these results, it is important to note that in our study, chemotherapy was linked exclusively to a reduction in the risk of OS-related deaths, without a corresponding decrease in CSS-related deaths. We hypothesize that this discrepancy may be due to selection bias; specifically, patients eligible for chemotherapy often exhibit a more favorable baseline health status and fewer comorbid conditions, which may decrease the likelihood of non-tumor-related mortality and consequently extend their OS. Additionally, baseline data from the IFCT-0501 study indicated that over 72% of enrolled patients had an Eastern Cooperative Oncology Group score of ≤1, suggesting that this selection bias may have contributed to the observed improvement in OS, thereby supporting our hypothesis. Unfortunately, the SEER database lacks certain variables, such as PS scores and underlying health conditions, which constrain our ability to further validate this conjecture. The poorer prognosis associated with patients exhibiting liver, brain, and bone metastases has been corroborated by multiple studies, including the findings of our own research. A prior observational study focusing on lung cancer identified the bone, brain, and liver as common sites for distant metastases. The median survival duration following diagnosis was reported to be 13 months for nonmetastatic patients, in contrast to only 5 months for those with metastatic disease. Specifically, patients with liver metastases had a median survival of approximately 3 months.^[33]^ Thus, distant metastasis serves as a significant prognostic factor in our model.

This study constructed a prognostic model based on a large sample cohort from the SEER database and validated it in an independent external cohort to assess its generalizability. The model integrates various clinical parameters such as age, sex, marital status, treatment methods, and distant metastasis status, offering a more refined individualized risk assessment compared with the traditional AJCC staging system, particularly for predicting the prognosis of elderly, nonsurgical NSCLC patients. In clinical practice, the model can estimate a patient’s 1-, 3-, and 5-year survival probabilities, assisting clinicians in risk stratification, treatment decisions, and follow-up planning. By identifying subgroups of patients who may benefit from active antitumor treatment, this tool helps balance treatment benefits and toxicity risks, offering more aggressive treatment strategies for fit elderly patients while avoiding overtreatment in those with poorer expected survival. Additionally, based on the model’s predicted survival probabilities, individualized follow-up plans can be developed, enhancing outpatient monitoring and symptom management for high-risk patients, and appropriately extending follow-up intervals for low-risk patients to optimize medical resource allocation. The model is easy to implement, making it particularly suitable for primary healthcare institutions lacking multidisciplinary treatment teams. The graphical presentation of the nomogram also facilitates communication and shared decision-making between doctors and patients. However, the external validation cohort has a limited sample size (n = 149), which may introduce uncertainty and optimism bias in the model’s performance evaluation. Although the external validation preliminarily supports the model’s applicability, the high AUC values (3- and 5-year OS of 0.940 and 0.915, respectively) should be interpreted with caution, and its generalizability requires further validation. Future research should conduct large-scale, multicenter external validation to assess the robustness of the model in diverse healthcare settings.

We utilized data from the SEER database in conjunction with an external cohort to develop a prognostic prediction model specifically designed for elderly patients with NSCLC who have not undergone surgical intervention. This population has not been systematically examined in prior research. Our model employs LASSO regression, which mitigates the impact of multicollinearity and enhances both the accuracy and applicability of the predictive model. Nevertheless, this model exhibits several limitations. First, we excluded a subset of patients due to the absence of critical information, which may introduce selection bias. Second, our model lacks certain prognostic factors potentially associated with NSCLC, including PS scores, family history, tumor markers, mutation status of driver genes, and data regarding immunotherapy and targeted therapies. With the widespread use of immune checkpoint inhibitors and targeted therapies, cancer treatment strategies have become increasingly diversified, and patient stratification management has grown more complex.^[34]^ One limitation of this study is that variables related to immunotherapy and targeted therapy were not included. Additionally, nutritional status and metabolic indicators are important prognostic factors that are not covered by this model. Existing studies indicate that baseline nutritional status is closely associated with response to antitumor therapy and patient tolerance.^[35,36]^ Malnutrition may affect treatment outcomes by influencing immune function and drug metabolism. Therefore, future model optimization should integrate key variables such as immunotherapy, targeted therapy, nutritional and metabolic indicators, and physical status scores to enhance prediction accuracy. Third, the SEER data do not provide detailed information on the reasons for nonsurgical treatment, which may include patients unable to undergo surgery for medical reasons, patients who actively refuse surgery, or those who cannot afford surgery due to socioeconomic factors, among other clinical circumstances. This heterogeneity is an inherent limitation of this study. The heterogeneity in the “nonsurgical” status could impact the model’s applicability in different healthcare systems. For instance, in regions with abundant healthcare resources, the proportion of nonsurgical patients may be lower and may reflect medical contraindications or patient choice. Conversely, in resource-limited regions, nonsurgical treatment may often indicate systemic barriers to healthcare access. Future multicenter studies should aim to collect detailed reasons for nonsurgical treatment (e.g., medical contraindications, patient refusal, resource limitations) to build more targeted prognostic stratification models. Fourth, this study used complete case analysis to handle missing data, which could introduce selection bias: patients excluded due to missing key information may have worse clinical characteristics (such as advanced age, advanced disease, or poor general condition), potentially biasing the analysis sample toward those with a better prognosis, thus overestimating the model’s predicted survival rate. Future research should employ multiple imputation methods to handle missing data in order to reduce selection bias and further assess the model’s generalizability through multicenter external validation. Finally, the external validation data utilized in our study were sourced from a single center; thus, multicenter external validation may further substantiate the applicability of this model in real-world settings.

## 5. Conclusion

This predictive model demonstrates enhanced accuracy in forecasting the prognosis of NSCLC patients aged 70 years and older who have not undergone surgical intervention. This may assist in clinical decision-making and prognostic assessment.

## Acknowledgments

We gratefully acknowledge the SEER public databases for providing the data platform, the data contributors, and the developers of the R software and related packages used for retrospective analysis in this study. We also extend our thanks to all those who have contributed to scientific research.

## Author contributions

**Conceptualization:** Feiyang Li.

**Methodology:** Feiyang Li.

**Data curation:** Fang Li.

**Resources:** Fang Li, Feiyang Li.

**Software:** Fang Li.

**Validation:** Fang Li, Haowei Lu.

**Visualization:** Fang Li, Haowei Lu.

**Supervision:** Haowei Lu.

**Project administration:** Feiyang Li.

**Writing – original draft:** Fang Li.

**Writing – review & editing:** Feiyang Li.
